# Leveraging Artificial Intelligence and Natural Language Processing in Legal Epidemiology Studies: Opportunities and Challenges

**DOI:** 10.1017/jme.2025.10224

**Published:** 2026

**Authors:** Regen Weber-Fares, Fallon Julia Cochlin, Snigdha Peddireddy, Mara Howard-Williams, Gregory Sunshine, Emily Ho, Cason Daniel Schmit

**Affiliations:** 1 Health Policy and Management, Texas A&M University School of Public Health, United States,; 2 Emory University, United States and Centers for Disease Control and Prevention, United States; 3 Public Health Infrastructure Center, Centers for Disease Control and Prevention, United States

**Keywords:** law, public health, artificial intelligence, legal epidemiology, natural language processing

## Abstract

Legal epidemiology — the study of how laws influence health outcomes — is an emerging field with potential to inform policy and improve public health. Shortages of specialists who can commit the extensive time required for these analyses has hindered progress in this area. Features of laws make them ideal candidates for artificial intelligence and natural language processing (AI/NLP) to address these constraints: standardized format, defined terms, and common terminology. However, there are concerns regarding valid AI/NLP application, especially given current underreporting of legal research in published policy evaluation studies. Drawing on lessons learned from case deployments and recent literature, we review opportunities and challenges for methods using AI/NLP in scientific legal research: assessing the scope of legal documents and data, identifying and collecting primary legal data, developing and applying coding schemes, and implementing quality controls to ensure the validity of produced legal datasets. We highlight methodologic research areas and innovation in the application of AI/NLP for scientific studies regarding effects of law on health and indicate methodologic reporting elements that will be essential for the field’s innovation. This review provides a method-focused research agenda for AI/NLP in scientific legal epidemiology studies that might accelerate the field’s growth and effects on evidence-based policy.

## Introduction

Unlike traditional legal research, legal epidemiology applies empirical methods to study effects of law on health.^1^ This process treats law as an exposure variable that must be reliably and validly measured to study its potential effect on health outcomes.^2^ With appropriate methodological application, legal epidemiology can be an invaluable tool to support evidence-based policymaking.^3^ Legal epidemiologic studies appear to be accelerating in academic publishing across disciplines, demonstrating broad interest and utility.^4^

The growth of the field of legal epidemiology is constrained by multiple factors, two of which are particularly significant in this context. First, legal epidemiology requires experienced interdisciplinary researchers trained in legal and empirical research methods. Researchers trained in both areas are uncommon, making it challenging to assemble legal epidemiology teams capable of bridging the two disciplines.^5^ Second, legal epidemiology studies tend to be demanding in terms of time and resources.^6^ For example, legal research in empirically crafted policy mapping and analysis studies can produce thousands of legal document results within the scope of the project, which require substantial time to adequately review for relevance.^7^ Additionally, longitudinal studies, which examine changes in law over time, require accurate collection of legal effective dates, or the dates which laws become legally enforceable, which often differs from the date the law is signed or passed.^8^ This adds a level of complexity and often requires additional training for researchers.^9^

The explosive developments in artificial intelligence (AI) and natural language processing (NLP) have significant and underexplored potential to address these constraints to the field of legal epidemiology. AI has provided powerful new tools to increase research efficiency and enable new discoveries. Machine learning methods have enabled systems to identify complex patterns in large-scale data that might be difficult or infeasible for humans to detect. In particular, NLP, or the ability of a machine to meaningfully and usefully “analyze, interpret and generate human language” in text and speech, has been a driving force of the generative AI boom.^10^ Early NLP approaches relied heavily on rule-based systems or frequency-based approaches (e.g., keyword searches). However, the field has rapidly evolved with advances in deep learning, particularly through the development of and application of large language models (LLMs), including the generative pretrained transformer (GPT).^11^ In theory, properly trained AI/NLP models can accelerate legal epidemiology research by automating tasks that are normally demanding, even for highly trained researchers.

However, the potential of AI/NLP comes at a precarious time in the field. Researchers have raised alarm that many studies purporting to evaluate the health effects of law are not transparent in their methods.^12^ The 2024 findings of Pepin and colleagues provided substance to many of these concerns.^13^ They reviewed 177 published studies and found frequent underreporting of methods used to measure law as an exposure variable in health studies. Among their findings, they discovered that 8% of studies failed to disclose any information on the source of the legal data. Additionally, 40% of published studies relied entirely on legal data from secondary source websites, which might not accurately display precise legal text.^14^ This review led to calls for reporting standards and published guidance for consumers of legal data or legal epidemiology studies.^15^

These methodological issues can potentially have policy implications. For example, an analysis by Carroll et al. highlighted fundamental errors in a previous longitudinal study of the impact of drug-induced homicide laws on rates of opioid overdose mortality.^16^ The authors argued that these flaws, including incorrect legal effective dates, undermine the primary conclusions of the study.^17^ Analyses with unreliable conclusions built on improper legal data have the potential to negatively impact public health policy if relied on by policymakers.^18^ These pervasive methodologic reporting concerns underscore the need for reviews of legal epidemiology methods and best practices.^19^

As AI/NLP technology rapidly advances, it presents new opportunities for public health researchers to elevate the standards of their public health law research practices, but risks exacerbating present concerns regarding methodological reporting. The opportunities and challenges presented using AI/NLP models in legal epidemiology research have yet to be fully discussed. This review seeks to outline the challenges and opportunities of AI/NLP in legal epidemiology drawn from the existing literature and early case studies of their use. Specifically, this review covers a method of collecting legal variables for a legal epidemiology study, or legal mapping. Legal mapping generally refers to the empirical process of creating legal data from primary legal sources (e.g., enacted legislation or promulgated regulations). Legal mapping methods consist of four phases, generally categorized as project scoping, legal research, coding, and quality control.^20^ This review describes multiple methodologic opportunities and challenges for using AI/NLP in each of these legal mapping phases.

### Current Literature and Early Deployment

No published studies have yet extended beyond AI/NLP-assisted legal text analysis to investigate the associations between coded provisions and population health outcomes. However, we can draw insights from the existing knowledge in other fields. For example, research in AI methods for traditional legal scholarship is more robust.

Scholarship in this area has focused extensively on AI’s potential use in legal practice. Investigations into the potential of AI/NLP to bolster the work of legal professionals have explored a range of traditional legalpractice areas with a range of results that highlight the potential applications for AI/NLP in legal research and practice but emphasize continued need for attorney oversight of AI/NLP use.^21^

A 2025 study evaluating multiple LLMs, including ChatGPT-4o and Claude 3.5 Sonnet, using legal practice guides to automate accurate answers to legal questions posed, reported mixed results.^22^ The authors reported that while providing excerpts from legal practice guides sometimes improved performance, the LLMs still struggled to answer legal questions. Across the tested areas of law, no model consistently outperformed the others.^23^ Because the study tested models that are now superseded by newer-generation LLMs, its findings should be interpreted in light of the rapid evolution of LLM capabilities.

A study of LLMs’ abilities to analyze tax law found that models provided with the correct legal text and proper prompting can accurately answer tax law questions, although not at the level of a trained tax attorney.^24^ Response accuracy improved with each iteration of the LLM.^25^ A 2024 study by Blair-Stanek et al. tested the ability of LLMs, such as GPT-4o and Claude-3.5 Sonnet, to handle legal texts that included deposition transcripts and statutory provisions.^26^ Although researchers reported these models performed poorly compared to what might be expected by users, they noted that with fine-tuning and training with a legal text dataset, the models were able to increase their accuracy to nearly 100%.^27^

Davoodi and Goldwasser’s 2024 study of US state legislative texts introduced a framework to normalize verbose, heterogeneous bill language into concise and standardized summaries using NLP.^28^ This work addresses concerns in harmonizing diverse statutory and regulatory texts across jurisdictions to facilitate comparison, a central challenge for legal epidemiology.^29^

Similarly, a recent evaluation explored whether LLMs such as GPT-3.5 and GPT-4 can automatically label portions of legal texts without needing a specially trained dataset.^30^ Researchers tested this zero-shot approach (i.e., where the model is simply given short descriptions of categories and asked to apply them without examples) using documents such as veterans’ appeals decisions, commercial contracts, and state statutes regarding public health emergencies. Importantly, GPT-4 performed almost as well as systems that had been trained on thousands of human-annotated examples. This study demonstrates how zero-shot models can meaningfully classify legal texts given only compact definitions, drastically lowering labor costs associated with manual annotation to subsets of datasets to ensure model performance matches expectations. This approach has the potential to democratize legal research, making workflows like contract review and policy surveillance accessible beyond highly trained and specialized legal teams.^31^ Since this study, further enhancements in LLMs have increased general model performance, which may solve previous flaws.^32^

Although literature exploring potential uses for AI in traditional legal practice is promising, it also highlights important considerations for researchers using AI tools. A 2024 study testing the legal knowledge of LLMs reported that these models frequently hallucinate, or return incorrect information.^33^ Hallucinations varied by level of court, jurisdiction, and the characteristics of the inputted question.^34^ Articles further emphasize the importance of human oversight of AI processes and caution users to understand limitations of AI methods in legal practice.^35^ The development of retrieval-augmented generation and other capabilities have mitigated some of the risk of hallucinations with real-time and domain-specific knowledge, but this risk is still present, particularly in fields which do not have hallucination benchmark studies.^36^

Additionally, there have been limited examples of these technologies applied to public health law research. The Public Health Adaptive Systems (PHASYS) project, a collaboration between CDC and the University of Pittsburgh, offers an instructive example.^37^ PHASYS researchers systematically coded state emergency preparedness statutes and applied network analysis methods to analyze and visualize agent relationships in state-level nuclear-radiological emergency response.^38^ Findings revealed substantial variation across states in the statutory design of preparedness systems, with certain jurisdictions embedding highly interconnected networks of authority, whereas others conferred powers more narrowly. Although PHASYS relied less on modern NLP methods than on systematic coding and computational and network analysis, it demonstrated how large-scale, structured legal datasets can explain the architecture and interconnectedness within public health systems. In this respect, PHASYS anticipated many of the aspirations of contemporary AI/NLP in legal epidemiology by transforming statutes into machine-readable data conducive to quantitative and comparative analysis.^39^

In addition to this existing literature, there have been efforts to build toward and even deploy the technology in public health and legal epidemiology applications. This paper will discuss a few of the most relevant efforts, with multiple authors having personal experience in these projects.

### CDC/ICF Emergency Public Health Orders

During the COVID-19 pandemic, the US Centers for Disease Control and Prevention (CDC), in partnership with ICF, developed a deep neural network to classify and tag state-level emergency public health orders collected by CDC legal epidemiology researchers.^40^ The collected laws were COVID-19 mitigation measures including mask mandates, vaccination mandates and prohibitions, and stay-at-home orders.^41^ This system processed tens of thousands of legal documents across more than 40 categories, saving the equivalent of more than 300 public health attorney workdays.^42^ By producing structured legal datasets at scale, this initiative transformed legal surveillance into a near-real-time process, allowing CDC researchers to devote more attention to evaluating effects of mitigation measures, such as mask mandates or business closures.^43^ CDC researchers were able to use these datasets to run legal epidemiology analyses with COVID-19 public health metrics, such as the impact of mask mandates on hospitalization rates or selfreported mask usage.^44^ In this application, AI/NLP greatly accelerated use of public health legal surveillance methods during an unfolding public health crisis. This type of rapid policy surveillance would have been impossible without these technologies.

### Temple University MonQcle

Researchers at Temple University’s Center for Public Health Law Research are actively testing an AI-assisted coding software embedded within the MonQcle legal coding platform.^45^ They see the potential of AI/NLP as tools to “augment human-generated legal research” rather than as a replacement.^46^ Specifically, they are exploring the potential for an “AI assistant” to supplement the quality control phase of a legal epidemiology project as a redundant coder. Their preliminary testing indicates that small variations in AI prompts can result in notable increases in coding accuracy (with some jurisdictions achieving over 90% accuracy benchmarked against human coders). Their initial methodological experiments that show how small prompt variations can result in significant changes in the accuracy of AI outputs illustrate the need for precise transparency in AI prompts to aid methodological learning across the field. Although this work is still in the early phases, their initial findings support the promise of AI/NLP in legal epidemiology and the need for methodology research to understand how these tools can be used transparently, reliably, and credibly.^47^

## Opportunities and Challenges in Legal Mapping Methods

Integration of AI/NLP into legal mapping studies has great potential but also raises serious concerns that demand discussion. First, we discuss certain broad opportunities and challenges for this application, before exploring the issue narrowly through each legal epidemiologic project stage.

The current landscape clearly shows that AI and NLP are promising tools for legal mapping. NLP’s systematic approach to document review, including automated provision classification, definition extraction, cross-reference identification, and open-ended text summarization, provides avenues to integrate these processes into the methodologic framework.^48^ Entirely text-based documents, statutes, regulations, and policy documents are often drafted within jurisdictions and provide a standardized format for AI/NLP analysis.

Legal codes provide strict definitions for important terms used within the legal document or chapter, which can anchor more precise NLP analyses. Further, LLMs have demonstrated improved capacity to capture long-range dependencies in text, which is particularly useful for handling the structured yet interdependent nature of statutes and regulations.^49^

Although legal text has certain advantages for AI/NLP, the application is fraught with challenges that should be carefully navigated. For example, the phenomenon of model drift poses challenges for AI applications. Model drift refers to a model’s decreasing performance over time, usually owing to novel applications or changing input-output dynamics.^50^ This is one reason why developers regularly release new models: to prevent model drift and mitigate knowledge cutoff errors. The rapid release of models raises additional concerns for research applications, though; when researchers use models that are rapidly replaced and retired, the replicability of research is further limited.

Another challenge is the dynamic and hierarchical structure of law. Statutes and rules often evolve through amendments, repeals, and administrative interpretations. This creates temporal complexity that complicates training data for AI/NLP models and longitudinal analysis. Jurisdictional variation adds further complexity, even where formatting and terminology are standardized within a single jurisdiction, the same term or concept can carry different meanings across jurisdictions, making interjurisdictional model comparisons difficult.^51^ Finally, concerns about fairness and transparency remain central, given the potential for AI/NLP models to encode existing biases present in training data.^52^ For example, if training data represents mostly jurisdictions with easily accessible laws, the AI/NLP model may learn patterns that reflectthose jurisdictions’ drafting norms, potentially leading to misclassification of the laws of underrepresented jurisdictions, such as local or tribal jurisdictions.

The following sections will explore the opportunities and challenges that arise from the application of AI/NLP in each of the four stages of a legal mapping study.

### Step I: Research Scope

The foundational step in a legal epidemiology research project is determining the project scope.^53^ To accurately collect and measure legal data, researchers must determine the jurisdictional and temporal scope of the laws, such as the jurisdictions, types of law, and period to be included. The type of legal and policy documents required for a project are specific to the health outcome variable being measured. This research stage requires the input of skilled subject matter experts in both the measured health outcome and the law.^54^

#### OPPORTUNITIES

Fundamentally, the research defining phase of legal epidemiology is driven by theory and hypotheses because it deals with basic methodologic questions (i.e., what will be studied and measured and why). For example, researchers should be able to explain why certain jurisdictions were included or excluded and why the study was limited to a certain period.^55^ Whether the field of legal epidemiology is ready for AI to shape or generate the theoretical models or hypotheses needed to guide the scoping phase of a specific study is unclear. AI/NLP has potential to aid human deliberations regarding the scope of a proposed legal mapping study and determinations related to whether a given document is within the scope of the research (e.g., brainstorming). However, it is likely that human experts will need to be closely involved in the final methodological decisions in this phase.

Opportunities are available to use AI/NLP to aid and guide these deliberations by identifying full breadth of existing legal data that might be included in a proposed legal epidemiology study. For example, legal epidemiology projects that investigate international or local jurisdictions face enormous scoping challenges because of jurisdictional differences in legal systems, structure of laws, and how legal documents are made publicly available.^56^ AI/NLP systems might be useful to inform human decisions about what types of laws could exist within a given study scope and which jurisdictions and types of laws are theoretically appropriate for study. For example, a researcher seeking to study the health impact of property zoning laws could use an AI/NLP model to determine a feasible scope by identifying the target jurisdictions, types of legal documents, or types of properties to be studied. Additionally, after scoping criteria are determined, AI/NLP systems might be used to aid determinations about whether collected laws meet those criteria. This process can have different levels of automation (e.g., using algorithmic confidence thresholds for automated vs. manual review). Researchers should attempt to assess the accuracy of the model on the given legal texts before using high levels of automation.

#### CHALLENGES

Deployment of AI/NLP in research scoping creates multiple challenges that should be addressed. The rationale for determining the scope of a legal epidemiology project should be transparent and scientifically sound, but this can be difficult to achieve with current AI/NLP models.^57^ Additionally, AI/NLP models are bound by the availability and accessibility of legal texts; many documents remain undigitized or are housed in databases with licensing restrictions. Even when available, models can either over- or under-include documents, depending on training data and methods used. Legal provisions often reference each other within the text of the provision, so a comprehensive legal database should include all necessary parts of a law, including other referenced laws and definitions, to avoid incorrect interpretation. Notably, the same legal terms can carry different meanings across subject areas or jurisdictions, so approaches that rely heavily on word similarity to identify relevant documents risk producing incomplete or incorrect samples of laws. This semantic variation can have ethical implications. For example, underrepresented jurisdictions’ laws may be more likely to have nonstandard terminology, potentially excluding these jurisdictions from the scope of research and the benefits of that research. Moreover, establishing research scope requires normative and theoretical judgments about which types of legal authority should be considered (e.g., statutory law vs. agency guidance vs. case law). AI/NLP methods cannot yet substitute for these interpretive decisions by subject matter experts.^58^

### Step II: Legal Research

This stage consists of identifying and collecting primary legal data. Typically, researchers will develop a search protocol using traditional legal databases and supplement this with secondary resources, such as official government websites or databases from previous legal research. All legal data is subsequently reviewed for relevance, quality, and completeness, which is the most time-intensive step in the research process. A legal search can return hundreds or thousands of texts across multiple sources and jurisdictions, all of which must be reviewed for relevance and accuracy.

#### OPPORTUNITIES

One major opportunity for AI/NLP in the legal research stage is improving legal document retrieval. A legal mapping study often requires collecting statutes, regulations, administrative orders, and guidance documents that are dispersed across multiple repositories and published in inconsistent formats, especially in local or international contexts. Modern NLP techniques (e.g., information retrieval or clustering) can automate the scanning of large collections of legal texts to flag potentially relevant documents. For example, models trained on a limited set of exemplar laws might identify candidate documents in other jurisdictions that use similar language, improving the efficiency of the retrieval process. Use of AI/NLP in legal document retrieval no longer remains purely theoretical, as traditional commercial legal research databases have begun integrating AI-assisted research features into their platforms to assist in legal research, analysis, and drafting (although many of the AI tools in legal databases were developed to aid traditional legal research and interpretation, which differs from the empirical approach of legal epidemiology).^59^

Beyond retrieval, AI/NLP models can also assist in de-duplication, identification of incorporated references, and normalization of metadata, all of which can streamline the harmonization of legal datasets. Harmonization is often one of the most labor-intensive parts of legal research, because laws from different jurisdictions can differ in formatting and terminology. AI/NLP can improve efficiency of this process by matching like terms, extracting elements like effective dates and cross-references, and detecting commonalities across jurisdictions. This can not only reduce manual review but also increase the reliability of comparisons across jurisdictions.

#### CHALLENGES

Not all laws or legally significant documents exist in formats immediately accessible to AI/NLP algorithms. Certain laws, particularly historical laws or categories of law such as executive orders, might only be available as scanned images rather than machine-readable text. To prepare these sources for AI/NLP analysis, researchers must use optical character recognition (OCR), another form of AI, to convert scanned PDFs into readable text. Legal documents pose unique challenges for OCR because all elements of their structure, including sections, subparagraphs, and citations, are crucial to analysis. Unlike other scanned documents, the importance of the inherent structure of legal documents creates additional considerations for researchers when converting documents using OCR. To preserve the structure, documents must first be converted into a proper format, then output as a file format which retains bounding box coordinates and other layout features. Consideration of document architecture becomes a crucial component of any automated approach to legal research.

The complexities of legal structures extend beyond formatting. Documents that might seem nonbinding, such as press releases or policy guidance documents, might inherit legal significance through associations with binding authority. For example, an apparently advisory guidance document becomes mandatory when incorporated by reference in a separate law. Identifying these associations can be difficult for AI/NLP and would likely require expert oversight. Although AI/NLP models might be trained to recognize and integrate these cross-references, they alone cannot make the determination of what constitutes law.

### Step III: Coding

After the primary legal research is complete, the next step is coding the laws. This involves extracting textual information and converting it into quantifiable data for analysis.^60^ Well-designed coding questions and structured rules for coding decisions are essential to building a reliable dataset with accurate variable measurement.

#### OPPORTUNITIES

Traditional machine learning models can be trained for simple classification, including specifically recognizing the presence or absence of key provisions (e.g., whether a statute mandates reporting). LLMs can go further, parsing complex language and categorizing statutes according to a multilabel coding scheme framed as prompts. Because laws often follow standardized formats and rely on recurring terminology, they can be well-suited to such automated pattern recognition. Automated coding can reduce duplication of efforts among research teams, improve reproducibility, and enable legal datasets to be updated at scale as new laws are enacted or amended.

AI/NLP also presents an opportunity to expand existing legal epidemiology datasets and protocols longitudinally through transfer learning.^61^ For example, a publicly available, cross-sectional legal dataset and protocol, in theory, provides a template set of instructions to train and instruct an AI/NLP model to replicate the protocol to historical laws, generating longitudinal data suitable for causal inference. This approach can be especially effective if the existing dataset links each coding determination to the specific legal text that supported it.

#### CHALLENGES

Coding requires a level of legal interpretation that poses challenges for AI/NLP models. Many provisions cannot be understood in isolation but depend on statutory definitions, related provisions, or external authorities. For instance, a statute might reference a controlled substance but define the term by reference to a separate schedule of drugs in another law. Ambiguity and conditional phrasing (e.g., “except as otherwise stated…” or “subject to…” clauses) add further complexity. Compiling training data for coding schemes is also labor-intensive, because creating annotated legal corpora requires extensive expert time and agreement on nuanced definitions.

Jurisdictional variations further challenge AI/NLP models. Key terms can differ not only from standard usage, but also across jurisdictions within the same project. For example, although a New York law referencing a “county judge” refers to the judiciary, the same term in Texas denotes a high-level county official.^62^ These contextual differences highlight the limitations of automating coding in legal mapping studies.

Additionally, methodologic research should include evaluation metrics such as interrater reliability to assess model performance against expert coders within the test dataset. Consistent with a “trust but verify” approach, a subset of the text should be coded by human experts to validate model accuracy and document potential errors (see discussion of redundant coding below). This process introduces additional complexity and resource demands to research but is necessary to align with best practices and to support transparency in legal epidemiology applications.^63^

### Step IV: Quality Control

High quality legal epidemiology projects implement quality control measures across all activities, including scoping, legal research, and coding. Quality control activities seek to ensure that the resulting legal data are complete, reliable, and valid by identifying potential errors in data or processes. Because these processes are fundamentally supplemental or supporting processes to the primary investigation, ample opportunities exist for AI/NLP systems to augment quality control.

#### OPPORTUNITIES

AI/NLP can support quality control in multiple ways throughout a study. At the research scoping stage, models can help ensure consistency in the chosen jurisdictional and temporal scope. During the legal research stage, AI/NLP can cross-reference existing research or secondary sources to identify gaps and facilitate greater dataset completeness. Within coding, AI/NLP might serve as a primary or secondary redundant coder. If the model is well-trained for this purpose, it can serve as an invaluable tool of quality checking in the coding stage. Finally, AI/NLP can be used to develop flagging protocols that automatically identify inconsistencies in coding or deviations from the established scheme.

#### CHALLENGES

Despite this potential, quality control is an area of legal mapping where limitations of AI/NLP are particularly pertinent. Models can produce erroneous or hallucinated results that might undermine the purpose of quality control.^64^ Because errors at this stage can compromise reliability, maintaining close human oversight is crucial. However, this raises a practical concern. If extensive review by experts is required to validate model output, process efficiency might not be improved and possibly may even be worsened by automation. Thus, a major challenge is to identify how AI/NLP can work as a supplemental tool without sacrificing rigor or efficiency in legal epidemiology.

Finally, this review highlights potential challenges in AI/NLP applications in all stages of the legal epidemiology process. Those challenges to AI/NLP-assisted scoping, legal research, coding, and quality control highlight the need for new quality control processes to address the novel issues raised by AI/NLP tools that would not otherwise be needed in human coding. For example, a researcher using an AI assistant as a non-human redundant coder may need a quality control process to verify that the AI assistant is an appropriate tool and sufficiently accurate to complete the task. Without additional research in the application of AI/NLP in legal epidemiology, it is unclear how much additional burden new quality control processes might be relative to the traditional manual approach.

## Reporting Considerations for Future Work

As a research method develops, reporting both legal epidemiologic and AI methods thoroughly to guide future work by researchers is important. Empirical legal researchers experimenting with AI/NLP models encounter unique challenges and novel validation requirements. Research with AI/NLP presents unique challenges that researchers must consider when reporting methodology. A particular challenge for AI/NLP research is the limitations of AI/NLP models in explaining decision-making processes.^65^ This is additionally pertinent as recent research suggests that generative AI use can undermine individuals’ perceived trustworthiness of the generated content.^66^ Additional research suggests increasing transparency increases trust of AI outputs.^67^ This underscores the importance of transparent reporting of AI methodology in legal epidemiology studies.

Previous explorations of methods reporting for empirical legal research have highlighted methodologic concerns rooted in improper methods reporting.^68^ As researchers continue to use AI/NLP in legal epidemiology, reporting methods used with precise clarity are particularly important. Until a research method has been adequately tested and the research community finalizes the methodologic standard, validating results as reliable will be difficult.^69^ To illustrate, whereas current generative models exhibit variability in outputs when asked the same question in slightly different ways, by accurately and consistently recording and reporting the prompts used, researchers will guide future legal epidemiology prompt engineering innovation and allow for prompt testing. Reporting methods at this early stage of AI/NLP use might require more extensive description in the supplemental materials of published papers. Ensuring validity requires transparent pipelines that document prompts, training data, decision rules, and other methodologic decisions that were fundamental to the study design.

## Areas for Future Research

This discussion reveals multiple opportunities for advancing research on the methodologic implications of using AI/NLP in legal epidemiology. Current literature and deployment efforts provide a solid foundation, but additional research is necessary to guide reliable, ethical, and scalable applications. Opportunities for future work can be organized into five key areas of focus: model development and benchmarking, prompt engineering, data infrastructure and access, methodologic innovation, and applications for public health practice.

### Model Development and Benchmarking

Future research can compare different AI/NLP models in legal mapping studies to assess appropriateness of their deployment. Different models might perform differently when parsing complex legal text, and performance can even differ between model versions.^70^ Systematic benchmarking against expertly validated datasets can enable researchers to evaluate reliability and identify areas of relative strength and weakness in the coding process. While there are currently no legal epidemiology benchmarks, future research could work to develop these benchmarks and maintain them over time. The development of large, open access annotated legal databases might further expand the possibilities for supervised learning, and explainability techniques might be explored for improving trust and reliability of automated coding.^71^ These future research opportunities can help establish a robust empirical foundation for AI/NLP deployment in legal mapping.

### Prompt Engineering

A key area for future research and development could aim to develop reliable AI/NLP prompts for legal epidemiology studies. It is broadly demonstrated that small changes in AI prompting can produce significant variation in the accuracy of outputs. This is demonstrated in the legal epidemiology field with the Temple University study discussed above. Prompt engineering enables the development of specific instructions to optimize AI/LLM outputs for specific tasks.^72^ While some initial learning will occur in clear methodology reporting, systematic and scientific prompt testing is needed to ensure accuracy and replicability in AI/NLP legal epidemiology outputs.

### Data Infrastructure and Access

Future research might also attempt to address the foundational challenge of legal data access. A great opportunity for this involves the development of an iterative, organized legal database that can collect legal documents from government websites, independent of traditional, paid legal databases. An AI model might facilitate automated web scraping, compiling and updating datasets to account for new or amended legislation. Incorporating introduced legislation might broaden surveillance to capture emerging policy concerns. Researchers can then use AI to sort laws into categories of relevancy, based on specific prompting. Such infrastructure might democratize access to legal data and improve the scalability and transparency of legal epidemiology.

### Methodologic Innovation in Legal Mapping

Legal mapping methods themselves might be reimagined with AI/NLP integration. Future work might test hybrid human-in-the-loop pipelines, where AI/NLP models perform first-pass coding that is subsequently reviewed and refined by legal experts. Researchers might also experiment with structured prompting strategies that might allow models to identify and apply definitional sections, trace legal cross-references, and reason step-by-step through the logic of provisions prior to coding. Machine learning can assist in capturing heterogeneity and complexity in ways that traditional methods cannot. Specifically, machine learning approaches can overcome certain limitations of traditional statistical modeling, including misspecification, multicollinearity, and overfitting, by flexibly modeling high-dimensional and unstructured textual data.^73^ Collectively, these innovations can expand the toolbox of legal mapping methods.

### Applications for Public Health Practice

AI/NLP in legal mapping might unlock novel applications that require further research. For example, extensions of CDC’s COVID-19 order pipeline might link categorized legal orders to epidemiologic indicators, including incidence, hospitalization, or mortality, enabling quasi-experimental policy analysis (e.g., using difference-in-differences designs). Real- or near-real-time surveillance might provide situational awareness for policymakers and practitioners. However, future research is needed to evaluate accuracy, timeliness, and updating processes. Researchers might investigate whether automated legislation tracking can be used to identify emerging health concerns not captured in traditional surveillance; because legislation often responds to salient social or health problems, introduced bills might offer early signs of public concern or lobbying activity. Such applications highlight the potential of AI/NLP-enabled legal epidemiology to address public health both retrospectively and prospectively, making them rich sources for future methodologic research.

Advancing AI/NLP in legal epidemiology will require interdisciplinary collaboration across law, public health, computer science, and ethics. By further investigating model development, data infrastructure, innovative methods, and applied use cases, researchers can improve legal mapping and deepen insights into the role of law in shaping population health.

## Conclusion

Applications of AI/NLP technologies offer unique opportunities and challenges for legal epidemiology, particularly within legal mapping methods. These advancements arrive at a crucial moment for empirical legal research, as the field faces deficiencies in methodology reporting and transparency. Legal mapping researchers must understand both the capabilities and limitations of AI/NLP tools. If used responsibly, these technologies using efficient document retrieval, relevancy scoping, and automated coding can reduce the labor intensity of legal epidemiology projects while expanding the breadth and quality of available data. By streamlining the review of large volumes of legal documents, AI/NLP can also improve accessibility, lowering barriers to legal epidemiology for organizations with limited resources.

Although there is great promise for these applications, challenges remain. Models can struggle with legal interpretation, jurisdictional variability, and complex legal structures. Careful human oversight and further investigations of limitations are essential to mitigate these risks. Looking forward, research designed at improving model performance, developing organized legal databases, and exploring novel legal research applications can further advance the utility of AI/NLP in legal epidemiology. We also stress the importance of reporting in this research and its applications, without which the field will struggle to converge on best practices. With careful consideration of relevant concerns, these efforts have the potential to strengthen methodologic quality, improve reproducibility, and contribute to greater effects of legal mapping on policy and public health interventions.
